# Supplementary material for: Maximum Entropy-Mediated Liquid-to-Solid Nucleation and Transition
Source: J Chem Theory Comput. 2025 Feb 12;21(4):1997–2011. doi: 10.1021/acs.jctc.4c01621 (PMC11866929; doi:10.1021/acs.jctc.4c01621)
Supplement: Supplementary file 1 — ct4c01621_si_001.pdf [file ct4c01621_si_001.pdf]

Supporting Information: Maximum entropy mediated liquid-to-solid nucleation and transition

Lars Dammann,*,†,‡,¶,§ Richard Kohns,¶,§ Patrick Huber,¶,§ and Robert H. Meißner*,†,‡

†*Institute of Surface Science, Helmholtz-Zentrum Hereon, 21502 Geesthacht, Germany*

‡*Institute for Soft Matter Modeling, Hamburg University of Technology, 21073 Hamburg, Germany*

¶*Institute for Materials and X-Ray Physics, Hamburg University of Technology, 21073 Hamburg, Germany*

§*Centre for X-ray and Nano Science CXNS, Deutsches Elektronen-Synchrotron DESY, 22607 Hamburg, Germany*

E-mail: lars.dammann@tuhh.de; robert.meissner@tuhh.de

Discrimination of phases with averaged local bond order parameters

The article presents several snapshots of biased simulations using average local bond order parameters to distinguish liquid phases from crystalline phases. The average local bond order parameters were developed by Lechner and Dellago[S1] and are a modification of the so-called local bond order parameters, also called Steinhardt order parameters.[S2] Steinhardt

order parameters are defined as

$$q_l(i) = \sqrt{\frac{4\pi}{2l+1} \sum_{m=-l}^{l} |q_{lm}(i)|^2}. \tag{S1}$$

with

$$q_{lm}(i) = \frac{1}{N_b(i)} \sum_{j=1}^{N_b(i)} Y_{lm}(\mathbf{r}_{ij}). \tag{S2}$$

Here $N_b(i)$ is the number of nearest neighbors to atom i. Y_{lm} are spherical harmonics with l being the degree and m the order of the spherical harmonic. $\mathbf{r}_{ij}$ is the connecting vector from particle i to particle j. The average local bond order parameters are obtained by introducing an additional averaging of the averaged spherical harmonic contributions to $q_{lm}(i)$ resulting in

$$\bar{q}_l(i) = \sqrt{\frac{4\pi}{2l+1} \sum_{m=-l}^{l} |\bar{q}_{lm}(i)|^2}, \tag{S3}$$

with

$$\bar{q}_{lm}(i) = \frac{1}{\tilde{N}_b(i)} \sum_{k=0}^{\tilde{N}_b(i)} q_{lm}(k). \tag{S4}$$

where the sum from $k = 0$ to $\tilde{N}_b(i)$ adds up the contribution from all nearest neighbors and the particle itself. $q_{lm}(k)$ is defined by Eq. (S2). In the simulation snapshots the average local bond order parameters $\bar{q}_6$ with a 3.5 Å cut off value are calculated for oxygen atoms to distinguish liquid water from hexagonal ice, to distinguish liquid TiO_2 from rutile $\bar{q}_8$ are calculated over the 12 nearest neighbors of Ti and to distinguish liquid TiO_2 from anatase $\bar{q}_{10}$ are calculated over the 12 nearest neighbor atoms of Ti. The respective thresholds of $\bar{q}_6 = 0.07$ for hexagonal ice, $\bar{q}_8 = 0.073$ for rutile, and $\bar{q}_{10} = 0.085$ for anatase were obtained by visual inspection of the histograms of the average bond order parameters at the beginning (unbiased) and end of the observed crystallization processes triggered by the biased simulations. Figure S1–S3 show the obtained histograms for the simulated transitions from liquid water to hexagonal ice, liquid TiO_2 to rutile, and liquid TiO_2 to anatase at the

start (blue) and end (red) of the biased simulations, respectively. The average local bond order parameters used to distinguish the liquid from the solid phases are marked by the black vertical line.

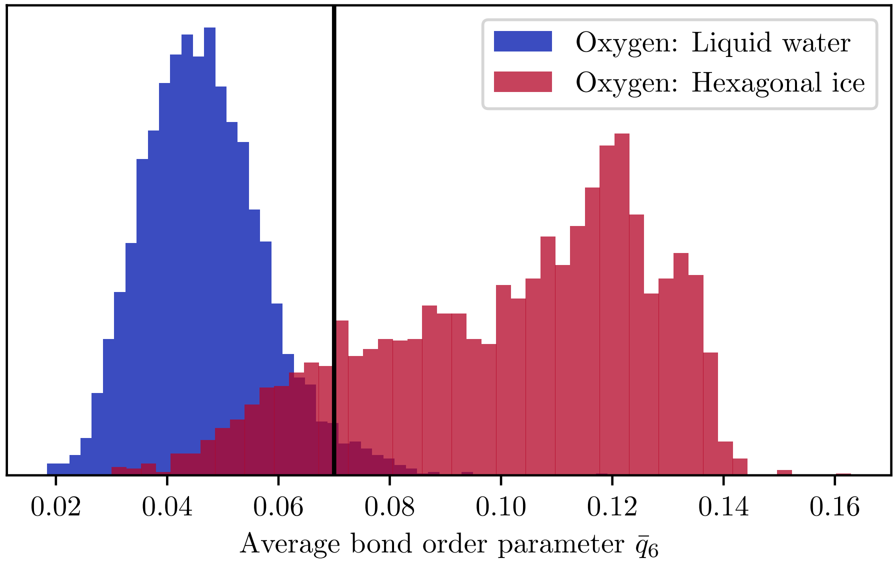

Figure S1: Histograms of the average local bond order parameters $\bar{q}_6$ calculated for oxygen atoms in unbiased liquid water (blue) and hexagonal ice created through biasing. The black line marks $\bar{q}_6 = 0.07$.

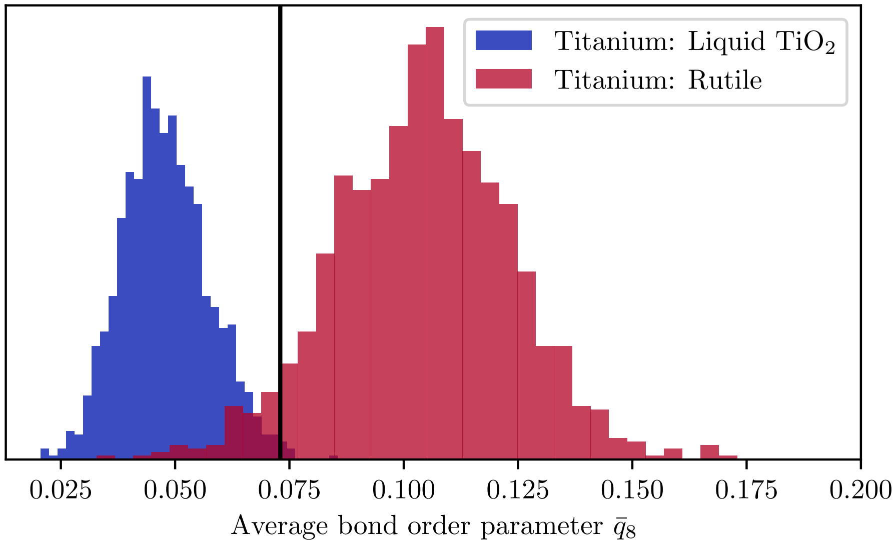

Figure S2: Histograms of the average local bond order parameters $\bar{q}_8$ calculated for titanium atoms in unbiased liquid TiO_2 (blue) and rutile created through biasing. The black line marks $\bar{q}_8 = 0.073$.

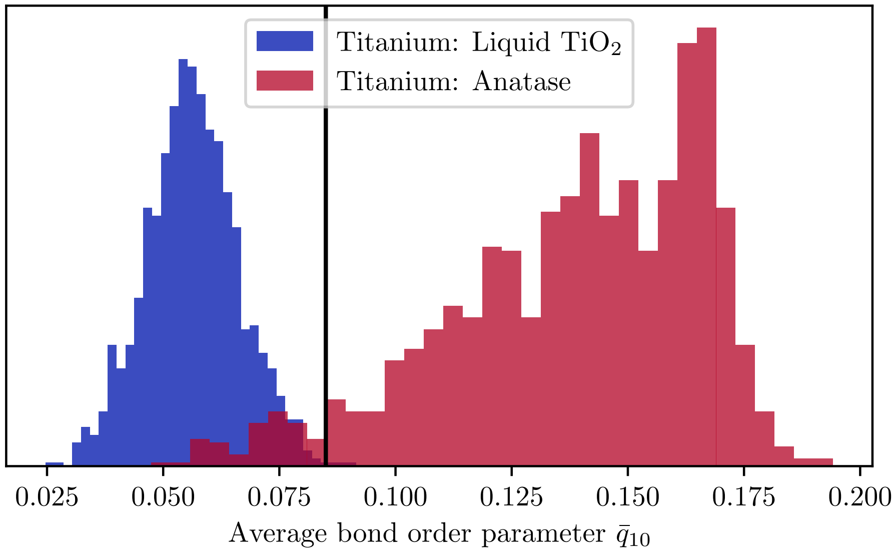

Figure S3: Histograms of the average local bond order parameters $\bar{q}_{10}$ calculated for titanium atoms in unbiased liquid TiO_2 (blue) and anatase created through biasing. The black line marks $\bar{q}_{10} = 0.085$.

Measurement of diffusion coefficients for oxygen in water molecules

The diffusion coefficients of the oxygen atoms in water were determined in two cases: for the simulated liquid water systems of the TIP4P/2005, TIP3P, and the biased TIP3P model, and for the gradually stronger biased liquid regime of the TIP4P/ICE model, prior to the crystallization transition. To this end, the mean squared displacement, $\langle S^2 \rangle$, of the oxygen atoms bound in the water molecules was determined in simulations. The diffusion coefficient, denoted by D, can be calculated using the linear relation

$$6DT = \langle S^2 \rangle \tag{S5}$$

where T is the time evolution of the system. To obtain a precise value for the diffusion coefficient, the slope of $\langle S^2 \rangle$ in dependence of time must be fitted. Fig. S4 depicts, the

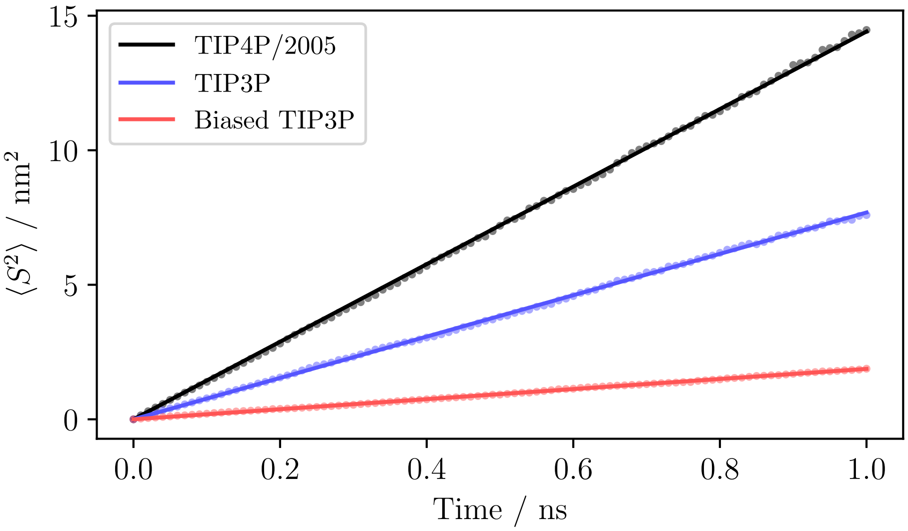

Figure S4: Mean squared displacement $\langle S^2 \rangle$ of the oxygen atoms in the water molecules in dependence of simulation time for the different water models at 300 K and 1 atm. The measured data is depicted as points and the linear fit as a continuous line.

measured mean squared displacement for the TIP4P/2005, the TIP3P and the biased TIP3P water model observed at 300 K and 1 atm. The reduction in diffusion for the biased TIP3P

model in comparison to the unbiased TIP3P model is clearly visible. Fig. S5 depicts the

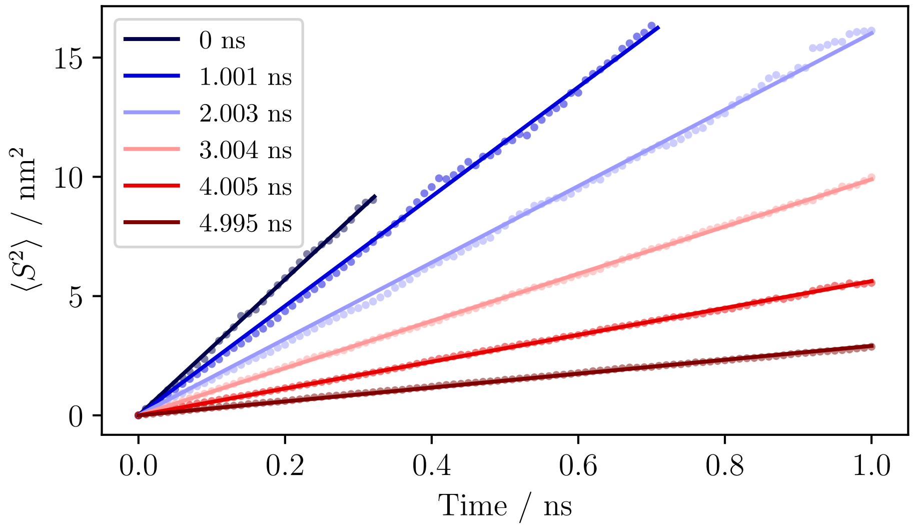

Figure S5: Mean squared displacement $\langle S^2 \rangle$ of the oxygen atoms in the water molecules in dependence of simulation time for the different for different bias strengths at 360 K and 1 atm. The measured data is depicted as points and the linear fit as a continuous line.

observed mean squared displacement for the TIP4P/ICE model at 360 K and 1 atm with bias strengths of the liquid regimes during different times of the simulated water-ice transition trajectory. The reduction in diffusion in dependence of the bias strength is clearly visible.

References

(S1) Lechner, W.; Dellago, C. Accurate determination of crystal structures based on averaged local bond order parameters. *The Journal of Chemical Physics* **2008**, *129*, 114707, DOI: 10.1063/1.2977970.

(S2) Steinhardt, P. J.; Nelson, D. R.; Ronchetti, M. Bond-orientational order in liquids and glasses. *Physical Review B* **1983**, *28*, 784–805, DOI: 10.1103/PhysRevB.28.784.
